# Social inequalities in tobacco-attributable mortality in Spain. The intersection between age, sex and educational level

**DOI:** 10.1371/journal.pone.0239866

**Published:** 2020-09-28

**Authors:** Mariana Haeberer, Inmaculada León-Gómez, Beatriz Pérez-Gómez, María Téllez-Plaza, Mónica Pérez-Ríos, Anna Schiaffino, Fernando Rodríguez-Artalejo, Iñaki Galán

**Affiliations:** 1 Departamento de Medicina Preventiva y Salud Pública, Universidad Autónoma de Madrid/IdiPAZ, Madrid, Spain; 2 Centro Nacional de Epidemiología, Instituto de Salud Carlos III, Madrid, Spain; 3 Centro de Investigación Biomédica en Red de Epidemiología y Salud Pública (CIBERESP), Madrid, Spain; 4 Departamento de Medicina Preventiva y Salud Pública, Universidad de Santiago de Compostela, Santiago de Compostela, Spain; 5 Direcció General de Planificació en Salut, Departament de Salut, Generalitat de Catalunya, Barcelona, Spain; 6 Institut Catala d’Oncologia, Hospitalet de Llobregat, Barcelona, Spain; Sciensano, BELGIUM

## Abstract

**Introduction:**

First study of social inequalities in tobacco-attributable mortality (TAM) in Spain considering the joint influence of sex, age, and education (intersectional perspective).

**Methods:**

Data on all deaths due to cancer, cardiometabolic and respiratory diseases among people aged ≥35 years in 2016 were obtained from the Spanish Statistical Office. TAM was calculated based on sex-, age- and education-specific smoking prevalence, and on sex-, age- and disease-specific relative risks of death for former and current smokers vs lifetime non-smokers. As inequality measures, the relative index of inequality (RII) and the slope index of inequality (SII) were calculated using Poisson regression. The RII is interpreted as the relative risk of mortality between the lowest and the highest educational level, and the SII as the absolute difference in mortality.

**Results:**

The crude TAM rate was 55 and 334 per 100,000 in women and men, respectively. Half of these deaths occurred among people with the lowest educational level (27% of the population). The RII for total mortality was 0.39 (95%CI: 0.35–0.42) in women and 1.61 (95%CI: 1.55–1.67) in men. The SII was -41 and 111 deaths per 100,000, respectively. Less-educated women aged <55 years and men (all ages) showed an increased mortality risk; nonetheless, less educated women aged ≥55 had a reduced risk.

**Conclusions:**

TAM is inversely associated with educational level in men and younger women, and directly associated with education in older women. This could be explained by different smoking patterns. Appropriate tobacco control policies should aim to reduce social inequalities in TAM.

## Introduction

Smoking, the most harmful health risk factor in Spain, led to 2,363 disability-adjusted life years per 100,000 people in 2016 [1]. According to National Health Surveys, one quarter of the Spanish population aged ≥15 years smoke. Though the prevalence has declined from 34.5% in 2001 to 25.5% in 2014, this decrease has been greater in men (42.2% in 2001 to 30.4% in 2014) than in women (27.3% to 20.5%, respectively). Specifically, declining trends are being observed in men of all ages but only in younger women, since among women aged ≥45 years the smoking prevalence is increasing [2].

Tobacco is also a leading contributor to health inequalities [3–12]. The unequal distribution of tobacco consumption is influenced by the social, economic, and environmental circumstances in which people are born, live, learn, work, and age (i.e., the social determinants of health) [13]. The theoretical framework of intersectionality [14] is being adopted by social epidemiology because it conceptually fits the model of social determination of health [15]. The framework states that there are multiple identities such as gender/sex and race/ethnicity (determinants) interlocked within an individual. These identities, far from being independent, interact and results into a multiplicative, rather than additive, effect on social oppression (harm to health); thus, they should be considered jointly. Therefore, the intersection between sex, age, and socioeconomic status—or its proxies such as educational level—may have a different effect on tobacco-attributable mortality (TAM) from what is observed in a separate analysis of each of them.

Social status influences tobacco use (prevalence) as well as other aspects of smoking, such as the type, frequency, and intensity of tobacco consumption, the age of initiation, the cessation rate (through motivation, demand, access to, and success of cessation therapies), and exposure to second-hand smoke [16]. Moreover, it may influence access to and quality of health care services, environmental exposure to other contaminants, and the presence of other health risks, including chronic biological stress, alcohol consumption, sedentary behavior, unhealthy diet, and morbidities [13, 17]. Overall, there is a social gradient and low socioeconomic status tends to have more of a negative impact than high status [3–12].

Some European studies that have examined social inequalities in mortality and survival attribute a substantial part of these inequalities to unequal tobacco consumption [3–12]. Gregoraci et al estimated that the contribution of smoking to socioeconomic inequalities in mortality varied between 19% and 55% in men and -1% and 56% in women [4]. Furthermore, Mackenbach et al identified smoking as the main contributor to the observed gap in partial life expectancy (19.8% in men and 18.9% in women) between the low- and highly-educated individuals aged 35–80 years. Thus, the potential for reducing the relative inequalities in premature mortality depends largely on lowering the prevalence of tobacco consumption in the groups with the lowest level of education [9]. At the regional level, absolute inequalities in TAM and the contribution of smoking to inequalities in total mortality in most countries have decreased among men, while increasing among women [4, 10]. Southern European countries, including Spain, are between the third and fourth stage of the tobacco epidemic. They have some of the smaller socioeconomic inequalities in TAM and tobacco use, especially among men and older adults. Whereas among older women the gradient is sometimes reversed, i.e., TAM and tobacco use being more common in higher socioeconomic groups [12, 18–21].

To our knowledge, studies assessing social inequalities in TAM for European populations have included data for just a few tobacco-attributable causes of death and from only three Spanish regions (Barcelona, Basque Country, and Madrid) which are not representative of the whole country. And of those national studies which have previously estimated the country-wide TAM in Spain [22–28], none assessed the joint influence of sex, age and educational level. Thus, this study aims to evaluate, for the first time in Spain, the hypothesis that there are social inequalities in TAM and that their direction and magnitude vary when analyzed from an intersectional perspective.

## Materials and methods

### Study design and population

Data were obtained from the Spanish Statistical Office, which provided cause of death, age, sex, and educational level for each deceased individual for the year 2016 (education data were not available prior to 2015) [29]. A total of 98.1% of deceased individuals aged ≥35 years had educational level data.

Tobacco-attributable causes of death were those listed in the Report of the Surgeon General [30]. The codes of the 10th-revision of the International Statistical Classification of Diseases (ICD-10) used included:

*Cancer*: malignant neoplasms of the lung, bronchus, trachea (C33-C34); lip, oral cavity, pharynx (C00-C14); esophagus (C15); stomach (C16); pancreas (C25); larynx (C32); cervix uteri (C53); kidney, renal pelvis (C64-C65); urinary bladder (C67); acute myeloid leukemia (C92.0); colon and rectum (C18-C20); and liver (C22).

*Cardiometabolic diseases*: ischemic heart diseases (I20-I25); other heart diseases (I00-I09, I26-I28, I30-I51); cerebrovascular diseases (I60-I69); atherosclerosis (I70); aortic aneurysm (I71); other vascular diseases (I72-I78); diabetes mellitus (E10-E14).

*Respiratory diseases*: influenza (J09-J11); pneumonia (J12-J18); tuberculosis (A15-A19); and chronic obstructive pulmonary disease (J40-J44).

TAM was calculated based on sex-, age-, and education-specific smoking prevalence, and on sex-, age- and disease-specific relative risks of death for current and former smokers vs lifetime non-smokers. The smoking prevalence was obtained by combining three health surveys (n = 66,673) in order to obtain more accurate estimates: the 2011 and 2016 National Health Surveys and the European Health Survey of Spain carried out in 2014 (S1 Table) [31–33]. The disease-specific relative risks were taken from five cohort studies: the National Institutes of Health-AARP Diet and Health Study, the American Cancer Society’s CPS-II Nutrition Cohort, the Women’s Health Initiative (WHI), the Nurses’ Health Study, and the Health Professionals Follow-Up Study (S2 Table) [30, 34].

The stratification variables were sex, age (35–54, 55–64, 65–74, ≥75 years) and educational level (low [up to primary/International Stardard Classification of Education (ISCED) 2011 [35] 0–1], medium-low [secondary/ISCED 2], medium-high [bachelor and tertiary/ISCED 3–5], high [university/ISCED 6–8]). All these variables are included in the mortality statistical database for each deceased individual. Denominators were obtained from population estimations developed by the Spanish Statistical Office [29].

### Statistical analysis

TAM was estimated as disease- and sex-specific tobacco population attributable fractions (PAF) by age group and educational level, and applying the PAF to disease-specific mortality data. First, PAF was calculated with the following formula:

[P1(RR1‐1)+P2(RR2‐1)]/[P1(RR1‐1)+P2(RR2‐1)+1]

where P1 is the prevalence of current smokers, P2 the prevalence of former smokers, RR1 the relative risk of death in current vs never smokers and RR2 the relative risk of death in former vs never smokers. Attributable deaths were calculated for each cause of mortality multiplying the observed mortality by the PAF. Second, crude TAM rates were calculated using the 2016 Spanish population denominators, and age-adjusted rates per 100,000 inhabitants were calculated by the direct method using the 2013 European standard population [36].

Finally, social health inequality indicators and their 95% confidence intervals (95% CI) were estimated based on adjusted rates. Absolute inequality was summarized by the slope index of inequality (SII). To calculate the SII, a weighted sample of the entire population was ranked from the less-educated subgroup (at rank 0) to the most-educated subgroup (at rank 1). This ranking was weighted, accounting for the proportional distribution of the population within each subgroup. The population of each subgroup was then considered in terms of its range in the cumulative population distribution, and the midpoint of this range (ridit). The adjusted mortality was then regressed against this midpoint value using a generalized linear model (Poisson distribution), and the predicted values of the mortality were calculated for the two extremes (rank 1 minus rank 0). SII is interpreted as the absolute difference in mortality risk between the lowest and the highest educational level, taking into account all intermediate values. If there is no inequality, SII takes the value zero. Greater absolute values indicate greater levels of inequality; positive values indicate a higher concentration of the mortality among the disadvantaged, while the opposite is true for negative values [37].

Relative inequality was summarized by the relative index of inequality (RII), a ratio of estimated values (rank 1/rank 0) of mortality for the less-educated over the most-educated, while taking into account the rest of subgroups. We calculated RII using the same procedure as for calculating the SII. RII is interpreted as a relative risk of mortality between the lowest and the highest educational level after accounting for all intermediate values. In the absence of inequality, RII takes the value 1. RII values >1 indicate a concentration of mortality among the disadvantaged and values <1 indicate the opposite [37]. Relative inequality was also represented by inequality concentration curves. These curves are computed by fitting (nonlinear optimization) a Lorenz concentration curve equation to the observed cumulative relative distributions of the population ranked by educational level and the cumulative mortality [38]. If the curve is below the 45-degrees line (the ‘equality’ line) the mortality is concentrated among the most-educated population, and if it is above such line, the mortality is concentrated among the less-educated population. The greater the area between the ‘equality’ line and the concentration curve, the greater the inequality.

Statistical analyses were performed in STATA v.15 (Stata Corp., Texas, US) for calculating TAM rates, and in HEAT Plus v.1.0 for estimating social health inequality measures [37].

## Results

In 2016, there were 53,436 deaths attributable to tobacco in Spain; 85% of them occurred in men (48% of the population), 50% among people with the lowest education (27% of the population), 25% in those with medium-low education (28% of the population), 15% in those with medium-high education (25% of the population), and 10% in those with the highest education (20% of the population). Cancer, cardiometabolic and respiratory diseases accounted for 50.7%, 27.0% and 22.2% of all deaths, respectively.

Tables 1 and 2 show the crude and adjusted TAM rates by disease, age and educational level in women and men, respectively. Total rates were higher in men: crude rates were 334 (men) and 55 (women), and adjusted rates were 235 and 43 per 100,000 men and women, respectively. This was true for each specific cause of death. The difference in adjusted mortality between men and women was greater in the older population (125 deaths per 100,000 in people aged ≥75 years) and in cancer mortality (52 deaths per 100,000). Overall, there was an inverse gradient between education and mortality in men (all ages and all causes) and in women aged 35–54 years (all causes). In women ≥55 years, a higher mortality rate was seen in the most-educated subgroups, although without a clear gradient since it was highest in medium-high than in highly educated women.

**Table 1:** Tobacco-attributable mortality in women according to educational level and age groups, Spain 2016.

Mortality by educational level	Total				35–54 years old				55–64 years old				65–74 years old				≥75 years old			
	Deaths	TAM	Crude rates	Adjusted rates	Deaths	TAM	Crude rates	Adjusted rates	Deaths	TAM	Crude rates	Adjusted rates	Deaths	TAM	Crude rates	Adjusted rates	Deaths	TAM	Crude rates	Adjusted rates
**All causes**																				
Low	61285	2957	67.2	28.6	472	172	22.1	6.2	1364	455	61.5	7.7	4391	664	59.7	6.3	55058	1667	94.0	8.5
Medium-low	15587	2341	59.6	39.6	971	411	21.3	6.0	1641	662	69.3	8.7	2273	502	81.6	8.6	10702	765	182.0	16.4
Medium-high	6551	1701	50.6	60.6	874	339	14.1	3.9	1007	486	86.8	10.9	1000	359	147.5	15.5	3670	517	337.3	30.4
High	4451	1104	36.6	50.4	501	161	7.3	2.0	690	329	69.1	8.6	757	294	143.0	15.0	2503	320	274.3	24.7
Total	87874	8103	55.1	43.3	2818	1084	14.8	4.1	4702	1932	70.7	8.8	8421	1818	83.6	8.8	71933	3268	132.7	11.9
**Cancer**																				
Low	11172	1124	25.5	13.4	268	88	11.4	3.2	757	291	39.4	4.9	1894	339	30.5	3.2	8253	405	22.9	2.1
Medium-low	4755	1243	31.7	19.6	598	245	12.6	3.5	1033	475	49.7	6.2	1184	311	50.6	5.3	1940	212	50.5	4.5
Medium-high	2576	972	28.9	29.3	592	220	9.1	2.6	717	385	68.8	8.6	546	217	89.3	9.4	721	150	97.8	8.8
High	1840	677	22.4	26.1	366	115	5.2	1.4	509	271	57.0	7.1	443	188	91.6	9.6	522	103	88.0	7.9
Total	20343	4016	27.3	21.3	1824	667	9.1	2.5	3016	1423	52.1	6.5	4067	1055	48.5	5.1	11436	870	35.3	3.2
**Cardiometabolic diseases**																				
Low	44740	1100	25.0	9.4	160	57	7.3	2.0	505	100	13.5	1.7	2220	209	18.8	2.0	41855	734	41.4	3.7
Medium-low	9605	733	18.7	13.2	324	134	6.9	1.9	512	122	12.8	1.6	939	121	19.6	2.1	7830	357	84.9	7.6
Medium-high	3449	490	14.6	20.3	251	99	4.1	1.2	244	67	12.0	1.5	376	84	34.7	3.6	2578	239	156.0	14.0
High	2233	269	8.9	14.7	122	39	1.8	0.5	153	38	8.0	1.0	251	62	30.1	3.2	1707	130	111.1	10.0
Total	60027	2592	17.6	14.0	857	329	4.5	1.3	1414	328	12.0	1.5	3786	476	21.9	2.3	53970	1460	59.3	5.3
**Respiratory diseases**																				
Low	5373	734	16.7	5.8	44	27	3.5	1.0	102	63	8.6	1.1	277	116	10.4	1.1	4950	527	29.8	2.7
Medium-low	1227	364	9.3	6.7	49	33	1.7	0.5	96	65	6.9	0.9	150	70	11.4	1.2	932	196	46.5	4.2
Medium-high	526	239	7.1	11.0	31	20	0.8	0.2	46	34	6.0	0.7	78	57	23.5	2.5	371	128	83.4	7.5
High	378	158	5.2	9.6	13	7	0.3	0.1	28	19	4.0	0.5	63	44	21.3	2.2	274	88	75.3	6.8
Total	7504	1495	10.2	8.0	137	88	1.2	0.3	272	182	6.6	0.8	568	287	13.2	1.4	6527	939	38.1	3.4

TAM: tobacco-attributable deaths. Crude and aged-adjusted tobacco-attributable mortality rates per 100,000 population.

Note: Cardiometabolic diseases include cardiovascular and cerebrovascular diseases and diabetes, Respiratory diseases include chronic obstructive pulmonary disease, pneumonia, influenza and tuberculosis.

**Table 2:** Tobacco-attributable mortality in men according to educational level and age groups, Spain 2016.

Mortality by educational level	Total				35–54 years old				55–64 years old				65–74 years old				≥75 years old			
	Deaths	TAM	Crude rates	Adjusted rates	Deaths	TAM	Crude rates	Adjusted rates	Deaths	TAM	Crude rates	Adjusted rates	Deaths	TAM	Crude rates	Adjusted rates	Deaths	TAM	Crude rates	Adjusted rates
**All causes**																				
Low	63053	23545	716.9	276.1	1267	637	68.7	19.2	3769	1977	333.1	41.6	10583	5410	688.9	72.3	47434	15521	1588.3	142.9
Medium-low	25854	11207	280.6	244.6	2629	1346	58.1	16.3	5037	2648	305.2	38.1	6481	3309	628.1	66.0	11707	3903	1380.9	124.3
Medium-high	14848	6392	175.8	226.0	1910	879	34.6	9.7	3150	1621	253.9	31.7	3620	1854	603.7	63.4	6168	2038	1347.1	121.2
High	10741	4189	158.2	181.5	716	260	15.2	4.3	1691	808	160.4	20.0	2690	1342	466.4	49.0	5644	1779	1202.8	108.3
Total	114496	45334	334.3	235.0	6522	3122	41.7	10.8	13647	7055	270.9	34.6	23374	11915	624.8	67.3	70953	23242	1490.7	136.8
**Cancer**																				
Low	21858	10806	329.0	133.7	606	307	33.1	9.3	2193	1205	203.0	25.4	5583	3238	412.3	43.3	13476	6056	619.7	55.8
Medium-low	11972	6257	156.7	129.8	1372	717	31.0	8.7	3161	1732	199.6	24.9	3682	2104	399.5	41.9	3757	1704	602.8	54.2
Medium-high	6971	3629	99.8	122.3	958	451	17.8	5.0	1918	1035	162.1	20.3	2052	1200	390.8	41.0	2043	943	622.9	56.1
High	4814	2397	90.5	98.6	394	147	8.6	2.4	1046	526	104.3	13.0	1569	893	310.4	32.6	1805	832	562.1	50.6
Total	45615	23089	170.3	122.7	3330	1622	21.7	5.6	8318	4498	172.7	22.0	12886	7436	389.9	41.7	21081	9534	611.5	55.3
**Cardiometabolic diseases**																				
Low	30866	5908	179.9	71.8	546	256	27.6	7.7	1310	548	92.3	11.5	3907	1321	168.2	17.7	25103	3783	387.1	34.8
Medium-low	11000	2971	74.4	62.6	1144	555	24.0	6.7	1605	687	79.1	9.9	2222	756	143.4	15.1	6029	973	344.2	31.0
Medium-high	6501	1844	50.7	60.8	883	386	15.2	4.3	1098	472	74.0	9.2	1294	446	145.3	15.3	3226	539	356.3	32.1
High	4884	1133	42.8	48.3	300	102	6.0	1.7	585	233	46.2	5.8	946	318	110.5	11.6	3053	481	325.1	29.3
Total	53251	11855	87.4	61.6	2873	1299	17.3	4.5	4598	1940	74.5	9.5	8369	2841	149.0	16.1	37411	5776	370.5	33.7
**Respiratory diseases**																				
Low	10329	6832	208.0	70.7	115	75	8.0	2.3	266	224	37.8	4.7	1093	851	108.4	11.4	8855	5682	581.4	52.3
Medium-low	2882	1979	49.5	52.2	113	73	3.2	0.9	271	230	26.5	3.3	577	449	85.2	8.9	1921	1227	433.9	39.1
Medium-high	1376	920	25.3	42.9	69	42	1.7	0.5	134	114	17.8	2.2	274	207	67.5	7.1	899	557	367.9	33.1
High	1043	659	24.9	34.6	22	11	0.7	0.2	60	50	9.9	1.2	175	131	45.5	4.8	786	467	315.6	28.4
Total	15630	10389	76.6	50.7	319	201	2.7	0.7	731	618	23.7	3.1	2119	1638	85.9	9.6	12461	7932	508.7	47.7

TAM: tobacco-attributable deaths. Crude and aged-adjusted tobacco-attributable mortality rates per 100,000 population.

Note: Cardiometabolic diseases include cardiovascular and cerebrovascular diseases and diabetes, Respiratory diseases include chronic obstructive pulmonary disease, pneumonia, influenza and tuberculosis.

Table 3 presents the social health inequality indicators by disease, sex and age. Among men, the less-educated had 61% greater risk of dying from tobacco-attributable diseases than the most-educated ones (111 additional deaths per 100,000 men). The increased relative risk was highest for respiratory diseases, followed by cardiometabolic diseases and cancer (2.5, 1.6 and 1.4, respectively); while the increased absolute risk was also higher for respiratory diseases, followed by cancer and cardiometabolic diseases (48, 37 and 27 additional deaths per 100,000, respectively). Among women, only the younger group (35–54 years) presented a similar inequality as men: overall, the less-educated had 95% higher mortality risk than the most-educated (3 additional deaths per 100,000 women). The increased relative risk was also slightly higher for respiratory diseases (based on very few cases) followed by cardiometabolic diseases (2.1-times higher risk). However, absolute inequalities were not statistically different for the individuals causes of death. In contrast, among women aged ≥55 years, the RII was <1 for overall mortality, indicating that the risk of tobacco-attributable death was lower in the less-educated (25% [55–64 years old], 73% [65–74 years old] and 84% [≥75 years old]) that in the most-educated subgroup. In total, there were 41 additional deaths per 100,000 women in the most-educated subgroups (specifically: 23 [≥75 years old], 12 [65–74 years old] and 3 [55–64 years old] additional deaths per 100,000 women). Among women aged ≥65 years, the increased relative and absolute risks were higher in cancer than in respiratory and cardiometabolic diseases. In women aged 55–64 years, the relative and absolute risks of cancer-related mortality were greater in the most-educated subgroup.

**Table 3:** Educational inequalities in tobacco-attributable mortality by sex and age groups. Spain, 2016.

Mortality cause	Age group	Women				Men			
		RII	95% CI	SII	95% CI	RII	95% CI	SII	95% CI
**All causes**	Total	0.39	0.35;0.42	-41.19	-45.79;-36.59	1.61	1.55;1.67	111.09	102.81;119.37
	35–54	1.95	1.65;2.25	3.20	2.62;3.78	6.28	5.97;6.58	19.18	18.39;19.97
	55–64	0.75	0.63;0.87	-2.52	-3.95;-1.10	2.17	1.99;2.36	26.33	23.36;29.30
	65–74	0.27	0.22;0.32	-11.59	-13.21;-9.97	1.50	1.40;1.60	26.63	22.23;31.03
	≥75	0.16	0.14;0.18	-22.93	-24.83;-21.02	1.45	1.37;1.52	49.70	42.46;56.93
**Cancer**	Total	0.36	0.31;0.40	-22.07	-25.23;-18.92	1.36	1.29;1.43	37.20	31.2;43.2
	35–54	1.24	0.93;1.54	0.64	-0.04;1.33	5.29	4.79;5.79	8.88	8.10;9.66
	55–64	0.55	0.44;0.65	-3.93	-5.17;-2.68	2.04	1.82;2.26	15.41	13.05;17.76
	65–74	0.19	0.15;0.24	-8.47	-9.76;-7.17	1.30	1.19;1.41	10.77	7.33;14.21
	≥75	0.11	0.08;0.14	-7.41	-8.45;-6.37	1.08	1.00;1.17	4.38	-0.08;8.85
**Cardiometabolic diseases**	Total	0.42	0.35;0.49	-12.26	-14.93;-9.58	1.55	1.43;1.66	26.78	22.49;31.07
	35–54	2.13	1.06;3.21	1.15	0.57;1.74	6.45	5.67;7.22	7.93	7.14;8.73
	55–64	1.65	1.00;2.30	0.75	0.15;1.34	2.00	1.68;2.33	6.48	4.93;8.02
	65–74	0.49	0.32;0.65	-1.66	-2.45;-0.88	1.58	1.36;1.79	7.15	4.99;9.31
	≥75	0.15	0.12;0.18	-10.44	-11.72;-9.16	1.27	1.14;1.41	8.03	4.49;11.57
**Respiratory diseases**	Total	0.43	0.34;0.53	-6.67	-8.66;-4.68	2.55	2.33;2.77	47.76	43.9;51.6
	35–54	58.72	-69.13;186.56	1.19	0.91;1.47	20.45	5.05;35.85	2.55	1.71;3.39
	55–64	2.24	1.04;3.45	0.67	0.21;1.13	4.56	3.19;5.93	4.53	3.55;5.51
	65–74	0.34	0.20;0.49	-1.48	-2.11;-0.86	2.70	2.19;3.20	8.98	7.22;10.75
	≥75	0.23	0.17;0.29	-5.13	-6.10;-4.16	2.33	2.11;2.56	39.15	34.52;43.79

CI: confidence interval, RII: relative index of inequality, SII: slope index of inequality.

**Fig 1:**
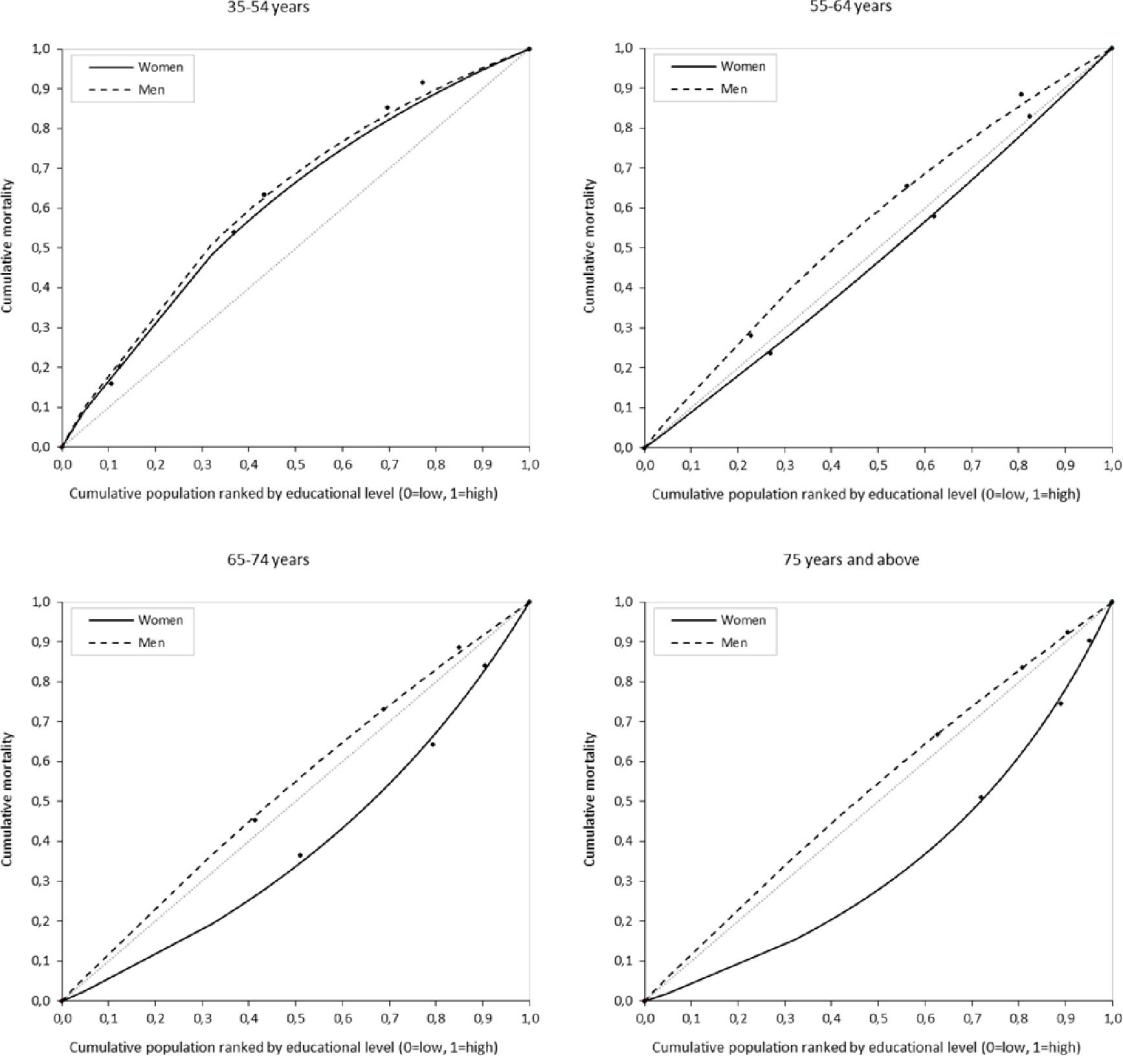
Inequality concentration curves in tobacco-attributable mortality according to educational level by sex and age groups. Spain, 2016.

Fig 1 shows the inequality concentration curves in TAM according to educational level by sex and age group. While among men the mortality is concentrated in the less-educated group in all ages (e.g., 50% of less-educated men [x axis] concentrate 69% of the deaths [y axis] in people aged 35–54 years and 55% of the deaths [y axis] in those aged ≥75 years), in women the mortality concentration varies by age: more concentrated among the less-educated for the 35–54 year-olds (curve above the equality line) but more concentrated in the most-educated group for the ≥55 group (curve below the equality line). Further, the highest inequality is seen in women aged ≥75 years, where 50% of most-educated (x axis) concentrate 72% of all deaths (y axis). Relative inequalities are higher in younger men (decreasing with age) and in the extreme age groups among women.

## Discussion

About 5 out of 6 tobacco-attributable deaths in Spain in 2016 occurred in men, and half of these deaths occurred among people with the lowest educational level (27% of the population), suggesting the presence of social inequalities in TAM. Further, the magnitude and direction of these inequalities vary by sex and age groups.

An inverse gradient between educational level and mortality was seen in men and younger women but, unlike what is reported in most high-income countries, not in women aged ≥55 years. In this age group, better social position (higher educational level) was associated with higher mortality and social inequalities increased with older age. These results are in line, at the national level, with several previous local studies in Spain [3, 18, 19, 23].

As TAM calculations are mainly driven by educational differences in smoking prevalence, the social inequalities in smoking-related mortality are consistent with the Spanish pattern of social inequalities in the prevalence. Among men, cohorts born after 1960 show higher smoking prevalence in lower levels of education. Among women, cohorts born between 1940 and 1980 show a direct educational gradient reaching the highest smoking prevalence in the highest educational groups, whereas cohorts born after 1980 present the same educational gradient as men, concentrating higher smoking rates in lower levels of education [39]. Besides, older women traditionally display higher differences in smoking prevalence according to social position when compared with middle-aged and younger cohorts. This reflects the smoking pattern that started in the later 1960s among the upper (literate) social group of women as a claim of social emancipation, modernity, and sexual equality. Afterwards, when prevalence rates started to fall, upper social class women did not quit smoking as much as their male counterparts of the same cohort [23, 40, 41]. Reasons for tobacco consumption among women include the linkage between smoking and the image of success and social relations-facilitator, and as a method to control stress and body weight. This is closely related with gender roles and social demands on women, including the overburden from both reproductive and paid work, and the socially-stereotyped corporal image. So, it is possible that in Spain older women of higher socioeconomic groups begin and remain smoking as a behavior to cope with greater social pressure [41, 42].

Social status influences not only tobacco use (prevalence), but also other aspects of smoking such as the type, frequency, and intensity of tobacco consumption, the age of initiation, the cessation rate, and exposure to second-hand smoke. A thorough review concluded that smoking prevalence is higher among lower socioeconomic groups, and that smokers from lower socioeconomic groups present with higher levels of cotinine concentration even when daily cigarette consumption is accounted for [43]. This suggests that they smoke each cigarette more deeply and/or frequently, and therefore extract more nicotine per cigarette. Also, among the disadvantaged groups, quit attempts are less likely to succeed, possibly due to reduced social support for quitting, lower motivation to quit, stronger nicotine addiction, lower likelihood of completing courses of pharmacotherapy or behavioral support sessions, reduced self-efficacy, and greater exposure/effects of tobacco industry marketing [16]. There are also some suggestions that, at least in some contexts, current tobacco control may be least effective among disadvantaged women [44–46]. One of the possible reasons why deprived people smoke more is that it is perceived as a way of managing stress, regulating mood and coping with everyday problems that occur due to adverse social circumstances. Moreover, while smoking may have become stigmatized in more affluent individuals, in lower socioeconomic groups smoking generally remains more tolerated. Smoking uptake occurs earlier in poor children whose parents, family and peers usually smoke or may consider smoking as being the norm or socially acceptable [47]. As a result, a substantial part of the educational inequalities in tobacco use are due to childhood characteristics shared by siblings [48], and to factors present in the adolescence, such as school attachment and having friends who smoke [40]. Furthermore, higher smoking rates among disadvantaged people lead to higher exposure to second-hand smoke [49].

Low socioeconomic status may also affect mortality directly, through increased anxiety and chronic biological stress [17, 50], and indirectly, whether through increasing other health risks such as alcohol consumption, unhealthy diet, sedentary behaviors and exposure to air pollution, or decreasing access or quality of medical care. Several studies have shown that smoking-related diseases have a steep social gradient that is only partially explained by smoking behavior—for example, smoking explains between 15–50% of the social differences in lung cancer [51–54] and about 15–17% of the social inequalities in cardiovascular mortality [47]. Low socioeconomic status is also an independent risk factor for all-cause mortality [55], cardiovascular morbidity [56] and mortality [57], as well as for lung cancer morbidity [51–54, 58] and mortality [59, 60]; even after adjusting for tobacco use and other lifestyles. With respect to the utilization of health care services, the National Health System in Spain provides universal coverage regardless of social or employment status, thus there is some evidence that the contribution to health inequalities is limited [61–63]. Nevertheless, compared to those with highest social status and/or additional private insurance, low socioeconomic groups and individuals relying on the National Health System do experience longer specialist waiting list times, less access to specialists, dentists and physiotherapists, and less access to preventive services (e.g., cervical cancer screening) which may influence mortality [64–66].

In theory, social inequalities in TAM may also be partly due to differential vulnerability to tobacco’s harm, i.e., same level of tobacco consumption could increase mortality due to tobacco’s interaction with low socioeconomic status [67]. Nevertheless, several studies have consistently reported no significant interaction between smoking and low socioeconomic status [34, 68–72]. Although Lewer et al reported in a large English cohort that smoking was associated with higher absolute lung cancer and chronic lung disease mortality risk in lower socioeconomic groups, even after accounting for the higher rates of smoking in these groups, this was due to higher baseline mortality rates among the disadvantaged people rather than to a negative interaction [73].

Among men and younger women, both absolute and relative inequalities were largest in respiratory diseases, supporting results reported by Kulik et al based on 18 European populations [5]. Among women 55 and over, both relative and absolute inequalities were larger in cancer than in other conditions. These results are driven by different TAM rates: higher incidence of cancer (accounting for half of all tobacco-attributable deaths) explains the absolute gaps, while the lower incidence of respiratory diseases (deaths due to chronic obstructive pulmonary disease, influenza, pneumonia and tuberculosis) among the most-educated population explains the relative gaps in men and younger women.

Smoking and its fatal consequences are determined not only by sex, but also by its interaction with age and social status. Awareness and monitoring of these social inequalities can help improving tobacco prevention and control strategies. Although smoking rates have been declining in many high-income countries, including Spain, declines have been slower or nonexistent among lower socioeconomic groups so that inequalities in smoking have increased [16]. In fact, some tobacco control policies, particularly individual or group-level smoking cessation interventions, appear to be more effective in higher socioeconomic groups [74]. Based on our results, men and younger women of lowest social status should be the target of preventive measures, but also older women of highest social position, because they are often forgotten in prevention strategies. Evidence of effective interventions among lower socioeconomic groups is scarce: raising the price of tobacco products appears to be the intervention with the most potential to reduce tobacco-related health inequalities, especially among the younger population. Targeted cessation programs and mass media campaigns may also contribute to closing these gaps [16]. Among older women, specific motivations for smoking should be assessed, especially those related with gender roles and related social demands. Moreover, tobacco control measures should also aim to improve the knowledge of female-specific pathogenic processes [40, 75–78]. Notwithstanding this, in order to reduce social inequalities in mortality, not only individual-level but wider social policies aiming at reducing socioeconomic disadvantages from a lifecourse perspective should be the overarching goal [13, 78, 79].

### Limitations

Due to the lack of historical nation-wide data on educational level of deceased individuals, trends in educational inequalities cannot be assessed. We have also used smoking prevalence and deaths occurring in the same calendar period, so the estimates do not account for the tobacco-related disease induction periods [30]. As a result, considering that the overall prevalence of tobacco consumption has decreased in Spain [2], TAM rates are surely underestimated. Another cause of underestimation of TAM is that our analyses do not include second-hand smoke-related mortality and deaths among young people. In addition, our estimates do not consider either the intensity or the lifetime smoking history, being the duration of exposure especially relevant for cancer risk [80].

A comprehensive description of additional limitations related to the methodology employed for estimating TAM can be found in the Report of the Surgeon General [30]. Because we obtained the relative risks from this source, a report based on US and not Spanish cohort data, and because these relative risks were not adjusted for education or other social position indicators, we cannot rule out potential ethnic and socioeconomic differences in vulnerability to tobacco. Notwithstanding this, as discussed before, previous work testing whether socioeconomic status modifies the association between smoking and health have found no significant interactions. Finally, confounding by other social determinants or individual risk factors could not be assessed due to data limitations. Nevertheless, data from a large cohort from the US National Health Interview Survey found that adjusting for race/ethnicity, alcohol consumption and adiposity had little effect on risk estimates [81].

## Conclusions

In Spain, TAM is inversely associated with educational level in men of all ages and women <55 years of age. However, in contrast to many high-income countries, TAM is directly associated with educational level in women aged 55 and over. Thus, our findings underscore the need for monitoring and accounting for social inequalities when designing, implementing, and evaluating national tobacco prevention and control strategies.

## Supporting information

S1 Table Prevalence of current and former smokers by educational level, sex and age groups. Spain, 2011–2016.
(DOCX)

S2 Table Relative risks by smoking status, disease, sex and age groups. (DOCX)

## Abbreviations

CI: confidence interval
RII: relative inequality index
SII: slope index of inequality
PAF: population attributable fraction
TAM: tobacco-attributable mortality
