# Supplementary material for: Social inequalities in tobacco-attributable mortality in Spain. The intersection between age, sex and educational level
Source: PLoS One. 2020 Sep 28;15(9):e0239866. doi: 10.1371/journal.pone.0239866 (PMC7521746; doi:10.1371/journal.pone.0239866)
Supplement: S1 Table — Spain, 2011–2016. (DOCX) [file pone.0239866.s001.docx]

**S1 Table. Prevalence of current and former smokers by educational level, sex and age groups. Spain, 2011-2016.**

|  |  | **Age 35-54** | | **55-64** | | **65-74** | | **≥75** | | |
| --- | --- | --- | --- | --- | --- | --- | --- | --- | --- | --- |
| **Educational level by sex** | **Smoking status** | **%** | **95%CI** | **%** | **95%CI** | **%** | **95%CI** | **%** | | **95%CI** |
|  |  |  |  |  |  |  |  |  | |  |
| **Women** |  |  |  |  |  |  |  |  | |  |
|  |  |  |  |  |  |  |  |  | |  |
| **Low** | Current smokers | 27.6 | 24.8;30.5 | 17.2 | 15.2;19.4 | 4.9 | 4.1;5.8 | 0.9 | 0.6;1.3 | |
|  | Former smokers | 17.6 | 15.2;20.2 | 15.6 | 13.7;17.6 | 9.3 | 8.2;10.6 | 3.5 | 3.0;4.1 | |
| **Medium-low** | Current smokers | 35.9 | 34.0;37.9 | 22.2 | 19.8;24.9 | 6.6 | 5.1;8.5 | 2.6 | 1.5;4.5 | |
|  | Former smokers | 20.9 | 19.3;22.5 | 22.0 | 19.5;24.6 | 13.2 | 10.9;16.0 | 8.7 | 6.4;11.8 | |
| **Medium-high** | Current smokers | 30.7 | 29.1;32.4 | 26.8 | 23.8;30.1 | 14.4 | 11.4;18.1 | 6.7 | 3.8;11.5 | |
|  | Former smokers | 23.9 | 22.4;25.4 | 29.9 | 26.8;33.2 | 23.1 | 18.6;28.3 | 15.4 | 10.7;21.8 | |
| **High** | Current smokers | 21.5 | 19.9;23.2 | 22.3 | 19.1;25.8 | 14.5 | 10.9;19.0 | 3.8 | 1.6;8.9 | |
|  | Former smokers | 26.7 | 25.0;28.4 | 37.4 | 33.4;41.6 | 29.5 | 24.4;35.1 | 17.0 | 11.4;24.6 | |
| **Men** |  |  |  |  |  |  |  |  |  | |
| **Low** | Current smokers | 43.6 | 40.5;46.8 | 28.3 | 25.7;30.9 | 17.4 | 15.6;19.4 | 8.6 | 7.4;9.9 | |
|  | Former smokers | 27.5 | 24.7;30.6 | 45.6 | 42.6;48.6 | 55.6 | 53.2;58.1 | 56.3 | 54.0;58.5 | |
| **Medium-low** | Current smokers | 43.5 | 41.7;45.4 | 29.8 | 26.9;32.9 | 18.4 | 15.1;22.3 | 10.4 | 7.2;14.9 | |
|  | Former smokers | 28.6 | 26.9;30.3 | 45.5 | 42.2;48.8 | 50.7 | 46.3;55.1 | 54.6 | 48.4;60.7 | |
| **Medium-high** | Current smokers | 34.8 | 33.1;36.5 | 30.6 | 27.6;33.9 | 18.0 | 14.7;21.8 | 11.8 | 8.3;16.7 | |
|  | Former smokers | 27.8 | 26.3;29.5 | 43.6 | 40.4;46.9 | 51.3 | 46.8;55.8 | 52.5 | 45.6;59.3 | |
| **High** | Current smokers | 20.6 | 18.9;22.4 | 25.5 | 22.0;29.3 | 15.9 | 12.6;19.8 | 8.1 | 5.2;12.3 | |
|  | Former smokers | 26.0 | 24.1;28.0 | 42.2 | 38.1;46.4 | 57.5 | 52.6;62.3 | 61.7 | 54.7;68.2 | |
| Source: Spanish National Health Surveys 2011 and 2016, and European Health Survey of Spain 2014 (n=66,673). | | | | | | | | | | |
