# Supplementary material for: Social inequalities in tobacco-attributable mortality in Spain. The intersection between age, sex and educational level
Source: PLoS One. 2020 Sep 28;15(9):e0239866. doi: 10.1371/journal.pone.0239866 (PMC7521746; doi:10.1371/journal.pone.0239866)
Supplement: S2 Table — (DOCX) [file pone.0239866.s002.docx]

**S2 Table. Relative risks by smoking status, disease, sex and age groups.**

|  | **Mortality cause** | **Women** | | | | **Men** | | | |
| --- | --- | --- | --- | --- | --- | --- | --- | --- | --- |
|  |  | **Age 35-54** | **55-64** | **65-74** | **≥75** | **35-54** | **55-64** | **65-74** | **≥75** |
| **Current smokers** | Malignant neoplasms of the lung, bronchus, trachea (C33-C34) | 13.30 | 18.95 | 23.65 | 23.08 | 14.33 | 19.03 | 28.29 | 22.51 |
|  | Other cancers* | 1.28 | 2.08 | 2.06 | 1.93 | 1.74 | 1.86 | 2.35 | 2.18 |
|  | Ischemic heart diseases (I20-I25) | 4.98 | 3.25 | 3.29 | 2.25 | 3.88 | 2.99 | 2.76 | 1.98 |
|  | Other heart diseases** | 2.44 | 1.98 | 1.85 | 1.75 | 2.40 | 2.51 | 2.22 | 1.66 |
|  | Cerebrovascular diseases (I60-I69) | 2.44 | 1.98 | 2.27 | 1.70 | 2.40 | 2.51 | 2.17 | 1.48 |
|  | Other vascular diseases*** | 2.44 | 1.98 | 6.81 | 5.77 | 2.40 | 2.51 | 7.25 | 4.93 |
|  | Diabetes mellitus (E10-E14) | 2.44 | 1.98 | 1.54 | 1.10 | 2.40 | 2.51 | 1.50 | 1.00 |
|  | Influenza (J09-J11), pneumonia (J12-J18), Tuberculosis (A15-A19) | 6.43 | 9.00 | 1.75 | 2.06 | 4.47 | 15.17 | 2.58 | 1.62 |
|  | Chronic obstructive pulmonary disease (J40-J44) | 6.43 | 9.00 | 38.89 | 20.96 | 4.47 | 15.17 | 29.69 | 23.01 |
| **Former smokers** | Malignant neoplasms of the lung, bronchus, trachea (C33-C34) | 2.64 | 5.00 | 6.80 | 6.38 | 4.40 | 4.57 | 7.79 | 6.46 |
|  | Other cancers* | 1.24 | 1.28 | 1.26 | 1.27 | 1.36 | 1.31 | 1.49 | 1.46 |
|  | Ischemic heart diseases (I20-I25) | 2.23 | 1.21 | 1.56 | 1.42 | 1.83 | 1.52 | 1.58 | 1.32 |
|  | Other heart diseases** | 1.00 | 1.10 | 1.29 | 1.32 | 1.07 | 1.51 | 1.32 | 1.15 |
|  | Cerebrovascular diseases (I60-I69) | 1.00 | 1.10 | 1.24 | 1.10 | 1.07 | 1.51 | 1.23 | 1.12 |
|  | Other vascular diseases*** | 1.00 | 1.10 | 2.26 | 2.02 | 1.07 | 1.51 | 2.20 | 1.72 |
|  | Diabetes mellitus (E10-E14) | 1.00 | 1.10 | 1.29 | 1.06 | 1.07 | 1.51 | 1.53 | 1.06 |
|  | Influenza (J09-J11), pneumonia (J12-J18), Tuberculosis (A16-A19), A15 | 1.85 | 4.84 | 1.28 | 1.21 | 2.22 | 3.98 | 1.62 | 1.42 |
|  | Chronic obstructive pulmonary disease (J40-J44) | 1.22 | 1.34 | 15.72 | 7.06 | 2.22 | 3.98 | 8.13 | 6.55 |
| *Other cancers include lip, oral cavity, pharynx (C00-C14); esophagus (C15); stomach (C16); pancreas (C25); larynx (C32); cervix uteri (C53); kidney, renal pelvis (C64-C66); urinary bladder (C67); acute myeloid leukemia (C92.0); colon and rectum (C18-C20) and liver (C22). | | | | | | | | | |
| **Other heart diseases include pulmonary heart disease (I26-I28), rheumatic heart disease (I00-I09) and other forms of heart diseases (I30-I51). | | | | | | | | | |
| ***Other vascular diseases include atherosclerosis (I70); aortic aneurysm (I71); other arterial disease (I72-I78). | | | | | | | | | |
| Source: United States Department of Health and Human Services. The Health Consequences of Smoking: 50 Years of Progress. A Report of the Surgeon General. Atlanta, GA: U.S. Department of Health and Human Services, Centers for Disease Control and Prevention, National Center for Chronic Disease Prevention and Health Promotion, Office on Smoking and Health, 2014. Printed with corrections, January 2014. | | | | | | | | | |
